# A New School, a Fresh Start? Change and Stability in Peer Relationships and Academic Performance in the Transition from Primary to Secondary School

**DOI:** 10.1007/s10964-024-01991-y

**Published:** 2024-05-04

**Authors:** Sofie J. Lorijn, Dieuwke Zwier, Lydia Laninga-Wijnen, Mark Huisman, René Veenstra

**Affiliations:** 1 Department of Sociology, University of Groningen, Groningen, the Netherlands; 2 Department of Sociology, University of Amsterdam, Amsterdam, the Netherlands; 3 Department of Developmental Psychology, INVEST flagship, University of Turku, Turku, Finland

**Keywords:** School transitions, Peer relationships, Academic performance, Attachment, Loneliness, Latent Change Score Models

## Abstract

Previous studies on peer relationships in school transitions neglected individual differences, or did not examine the relation with academic performance in secondary school. This study followed 649 students from their last year of primary school to their first year in secondary school (*M*_*age*_ at T1 = 11.6 (*SD* = 0.6); 53.6% girls). Results revealed that students became more attached to peers, less lonely, and were stable in victimization across the transition. Particularly students with more negative peer experiences in primary school enjoyed a “fresh start” in terms of peer experiences in secondary school. Students who had more co-transitioning peers experienced greater reductions in loneliness. Changes in peer experiences over the transition did not relate to academic performance in secondary school.

## Introduction

School transitions pose significant challenges for students. Whereas primary schools are confined and familiar, secondary schools are larger, often further away from home and students are confronted with new teachers, an unfamiliar peer group, and higher educational expectations. Out of all changes, students worry most about their peer relationships as they transition to secondary school (Jindal‐Snape et al., 2020). In addition to social challenges, students face academic challenges when transitioning to secondary education. Previous studies confirm students’ concerns, generally demonstrating adverse effects of school transitions on students’ peer relationships and academic outcomes (Jindal‐Snape et al., 2020). At the same time, the transition to a new peer group is viewed as a potential “fresh start” in terms of peer relationships, especially for students with more negative peer experiences in primary school (Kinney, 1993). More insight into this topic is needed for two main reasons. First, previous studies have predominantly examined average changes in peer relationships over school transitions, neglecting heterogeneity in transition experiences. Particularly, students’ social position before the transition and the extent to which the peer group changes may explain heterogeneity in transition experiences. Second, whereas some previous research examined both social and academic outcomes in school transitions and found these were related (Felmlee et al., 2018; Lessard & Juvonen, 2022), previous studies overlooked whether changes in peer experiences predict academic performance. This study examined to what extent attachment, loneliness, and victimization (dis)continued over the transition from primary to secondary school. Beyond studying average changes, this study investigated to what extent (1) students’ attachment, loneliness, and victimization in primary school relate to the (dis)continuation of these peer experiences over the transition from primary to secondary school, (2) the proportion of co-transitioning peers relates to this (dis)continuation, and (3) this (dis)continuation relates to academic performance in secondary school.

### (Dis)continuation of Peer Experiences across School Transitions

Many students worry about losing their primary school friends and having difficulty fitting into the new peer group in secondary school (Jindal‐Snape et al., 2020). Establishing positive peer relationships and avoiding negative peer experiences is particularly important for adolescents given their heightened desire to establish and maintain peer relationships (Veenstra & Laninga-Wijnen, 2023). Key aspects of students’ peer experiences include peer attachment, loneliness, and victimization. Students’ attachment to peers is generally defined as sharing a deep and enduring emotional bond (Bagwell & Bukowski, 2018; Bowlby, 1973). In the school context, peer attachment for instance reflects feelings of acceptance and being understood by classmates. Loneliness is the unpleasant feeling that occurs when there is a discrepancy between a person’s perceived and desired quantity or quality of social relationships (Perlman & Peplau, 1981). Considering the importance of peers for adolescents, peer-related loneliness may be particularly detrimental. At school, feeling lonely among peers for instance entails feeling excluded by classmates. Victimization refers to a student repeatedly being exposed to goal-directed aggressive actions by one or more classmates in the context of a power imbalance (Olweus, 1993). These aggressive actions can take different forms, including physical, relational, material, and cyber-victimization. Higher levels of attachment have been found to relate to lower levels of loneliness and victimization, whereas loneliness and victimization are generally positively related (Lorijn et al., 2023).

For many students, peer experiences may continue over the transition from primary to secondary school. For instance, one study found that students’ loneliness remained relatively stable across the transition (Kingery et al., 2011). Students were also relatively stable in psychological well-being (Virtanen et al., 2019), school adjustment, and psychological adjustment (De Moor & Branje, 2023) across the transition. Students who were more accepted by peers and had more and higher quality friendships before the transition, were less lonely after the transition (Kingery et al., 2011), suggesting that positive peer experiences continue for some students. Making new friends in secondary school was especially difficult for students with negative peer experiences such as victimization in primary school (Evangelou et al., 2008), suggesting that negative peer experiences continue for at least some students. Continuation of peer experiences fits with attachment theories arguing that social relationships are relatively stable, as early attachment relationships set the stage for later attachment relationships (Bowlby, 1973). Thus, on average, continuation in students’ levels of attachment, loneliness, and peer victimization is likely to occur.

Nevertheless, for some students, peer experiences may discontinue over the transition from primary to secondary school. In a recent review on school transitions, ten studies reported a negative impact of the transition on their peer experiences, whereas seven studies reported an improvement in peer experiences (Jindal‐Snape et al., 2020). For instance, in contrast to the previous mentioned study showing stability in loneliness (Kingery et al., 2011), other studies found that loneliness increased following a school transition (Benner et al., 2017; Benner & Graham, 2009), decreased (Lorijn et al., 2023), or increased or decreased depending on the school transition (i.e., elementary to middle school vs. middle to high school; Barber & Olsen, 2004). Moreover, whereas most students who structurally transitioned to high school in the U.S.A. were more likely to have fewer friends and become isolated than students who did not transition, some students gained friends (Felmlee et al., 2018; Temkin et al., 2018). Other studies highlighted that adolescents who transitioned schools improved their social status in the new classroom and gained friendships (Kinney, 1993; Symonds & Hargreaves, 2016). Discontinuation of peer experiences fits life-course theories, which argue that life transitions such as school transitions can greatly impact social relationships (Almeida & Wong, 2009).

The stage-environment fit theory (Eccles et al., 1997) provides a possible explanation for improvements or deteriorations in peer experiences across school transitions. A mismatch between adolescents’ needs and the opportunities provided by the school environment may result in lower social adjustment. Conversely, a match between adolescents’ needs and the opportunities provided by the school may improve adolescents’ social adjustment. When the school environment remains similar, stability in peer experiences may be expected. In terms of peer relationships, adolescents have an increased need for autonomy from their parents and seek closer peer relationships (Veenstra & Laninga-Wijnen, 2023). For some adolescents, the secondary school environment does not meet these needs, as their primary school peer network may have been disrupted and it may be challenging to fit into the new secondary school peer group. These youth may experience decreased social adjustment following the transition. For other adolescents, however, the secondary school environment may provide a better stage-environment fit. For these adolescents, the transition to a new peer group provides an opportunity to improve their peer status and develop friendships with a wider range of peers. In sum, depending on students’ stage-environment fit, students’ peer experiences may continue, weaken, or improve across school transitions. Given these differences between students, it is important to examine predictors for (dis)continuation of peer relationships.

### Primary School Peer Experiences

The extent to which students’ peer experiences change over the transition from primary to secondary school, may depend on their peer experiences in primary school. Possibly, the prevalent negative effects of school transitions on students’ peer relationships, as evidenced in prior research (Benner et al., 2017; Felmlee et al., 2018), specifically pertain to the majority of students who had positive peer relationships in primary school. Students who are high in the social hierarchy in primary school, socially have more to lose when entering a new peer group. For these students it may be most challenging to regain their favorable social position or even improve their position. Students with negative peer experiences in primary school may benefit from school transitions. For these students, the transition is an opportunity to establish new peer relationships from a wider group of peers, and enhance their social status in the new group (Jindal‐Snape et al., 2020). For instance, particularly students with fewer friends compared to their peers in primary education, gained friends after the transition to secondary education (Felmlee et al., 2018). Moreover, for a majority of students who were victimized in primary school, victimization did *not* persist in secondary school (Låftman et al., 2024; Pellegrini et al., 2010). More diverse subgroups of students in secondary school give students who previously felt left out an opportunity to fit into the peer group (Kinney, 1993). Possibly, school transitions lead to a reorganization of the social hierarchy in the classroom. This may be less beneficial for students with more positive peer experiences in primary school as they may be less likely to socially gain from transitioning to a new peer group, but more beneficial for students with more negative peer experiences in primary school.

### Co-transitioning Peers

Students’ peer experiences may particularly be subject to change when they enter a new, unfamiliar peer group in secondary school (Temkin et al., 2018). For example, decreases in victimization following a school transition were likely to be caused by the reorganization of the social hierarchy (Farmer et al., 2011). Conversely, when more primary school peers co-transition to the same secondary school, students’ peer attachment, loneliness, and victimization are more likely to continue. For instance, students kept the same friends if they made the same transition as their classmates from primary school, whereas friendships were more often disrupted when students transitioned to a new peer group (Temkin et al., 2018). Students may even choose the same secondary schools as their peers to maintain these relationships and benefit from ‘transitional support’ in this challenging phase (Zwier et al., 2023). Adolescents’ desire to continue positive peer experiences across school transitions may be explained by significant neurobiological changes that increase the importance of belonging to the peer group (Veenstra & Laninga-Wijnen, 2023). Thus, students who are attached to their classmates in primary school and who enter a new school with more primary school peers, are more likely to continue these attachment relationships. Students who were lonely in primary school and transition with many familiar peers may have continued experiences of exclusion and have less opportunity to form new connections. Students who were victimized in primary school and transition to secondary school may be confronted with a similar social hierarchy where they find themselves at the bottom.

### Academic Consequences of Changing Peer Experiences

Social integration in the peer group is widely acknowledged to be crucial for students’ academic development (Deci & Ryan, 2008). Peers in class can provide students with social support, act as socializing agents, and provide a context wherein a hierarchy is established (Ryan & Shin, 2018). Receiving more social support, academic socialization, and having a high status in class fosters students’ academic development. Students who are more accepted by classmates generally achieve academically higher (Wentzel et al., 2021). These students may like school more, have higher self-efficacy, and be more engaged and motivated in school (Kiuru et al., 2020; Wentzel et al., 2021). Instead, students who are victimized or lonely encounter heightened educational difficulties (Heinrich & Gullone, 2006; Kretschmer et al., 2018). These students may experience the classroom as a socially unsafe space to learn (Schacter et al., 2022), and struggle with lower self-esteem, self-efficacy, and motivation.

(Dis)continuing peer relationships across school transitions are likely to relate to students’ academic performance in secondary school. For instance, maintenance of friendships across a school transition promotes school adjustment in secondary school (Lessard & Juvonen, 2022). Moreover, establishing a wider range of friendships in the new peer group after a school transition stimulated school engagement (Symonds & Hargreaves, 2016). Following this reasoning, students who experience an increase in attachment or a decrease in loneliness or victimization across the transition may enjoy a fresh start not only socially, but also academically. Being more attached to classmates in secondary school may boost students’ self-esteem and school well-being (Kinney, 1993), making students academically flourish (Wentzel et al., 2021). Conversely, students who experience an increase in loneliness or victimization in secondary school may perform less well academically. Students who were not lonely or victimized in primary school but experienced this for the first time in secondary school may perform lower as a consequence of failing to re-establish their favorable position in the peer group. These students likely anticipated that they would re-establish their positive social position yet were disappointed by having more negative peer experiences in secondary school, negatively affecting their self-esteem (Poorthuis et al., 2014) and academic performance. Students whose loneliness or victimization persisted may also perform academically lower. Failure to improve their social position may cause an additional setback to their self-esteem and well-being as these students may attribute the negative peer experiences in different contexts to themselves (‘it must be me’) rather than to others (‘it could be them’; Huitsing et al., 2012). These factors, in turn, may adversely impact students’ academic outcomes.

## Current Study

Prior studies neglected individual differences in changing peer experiences across school transitions, or did not examine the relation between changes in peer experiences and academic performance in secondary school. The current study examined the persistence or change in attachment, loneliness, and victimization across the transition from primary to secondary school. Beyond average changes, this study examined (1) the relation between students’ peer experiences in primary school and the (dis)continuation of peer experiences across the transition, (2) the effect of the proportion of co-transitioning peers on this (dis)continuation, and (3) the extent to which this (dis)continuation is related to academic performance in secondary school. It was explored to what extent students’ average attachment, loneliness, and victimization (dis)continue over the transition from primary to secondary school. The expectations for this study revolved around three main hypotheses. First, it was expected that students who were more attached, lonely, or victimized in primary school, would decrease more (or increase less) in attachment *(Hypothesis 1A)*, loneliness *(Hypothesis 1B)*, or victimization *(Hypothesis 1C)*. Second, it was expected that the presence of more co-transitioning peers would be related to less change in attachment *(Hypothesis 2A)*, loneliness *(Hypothesis 2B)*, and victimization *(Hypothesis 2C)* from primary to secondary school. Third, the hypotheses extended to academic performance. It was expected that students who increase more (or decrease less) in attachment would perform better academically than students who increase less (or decrease more) in attachment *(Hypothesis 3A)*, and it was expected that students who increase more (or decrease less) in loneliness or victimization would perform worse academically than students who increase less (or decrease more) in loneliness *(Hypothesis 3B)* or victimization *(Hypothesis 3C)*.

## Methods

### Procedure and Participants

Data from the PRIMS project (an acronym for transition from PRIMary to Secondary school, for more information see Zwier et al., 2023) and administrative data from the Netherlands Cohort Study on Education (NCO; for more information, see Haelermans et al., 2020) were used. PRIMS aimed to study the role of peers in the transition from primary to secondary education in the Netherlands, and followed participants over this transition. This study included data from the second and third waves. The primary school measure (T1) was conducted in May and June 2021, during students’ final months in primary education. The secondary school measure (T2) was collected in January and February 2022, roughly five months after the students transitioned to secondary education. Compulsory education in the Netherlands consists of primary and secondary education. Students attend primary school for eight years, roughly from age four to age twelve, after which they transition to secondary school. The Dutch secondary school system is highly stratified: students are allocated to different tracks based on their prior performance and follow different curricula, often in separate schools. Secondary education consists of three main tracks: the pre-vocational track, the senior general track, and the pre-university track.

Full-population administrative data show that, in 2021, approximately 41% of all students were recommended for a pre-vocational track, 27% for a senior general track, and 32% for a pre-university track. In the sample matched to administrative data (N = 633), students in the highest tracks are overrepresented: 27.9% of students were recommended for a pre-vocational track, 28.7% for a senior general track and 43.3% for a pre-university track. While this quasi-binding track recommendation constrains which secondary schools students can choose from, students otherwise have considerable freedom in school choice (Zwier et al., 2023). Virtually all schools are publicly funded, students are not geographically bound to a particular school, and admission lotteries are uncommon. Due to the high population density, most students have several secondary schools to choose from, with an average of eight schools to choose from (*SD* = 6) within close proximity to home (Zwier et al., 2023). In addition to track offer, secondary schools differ in religious denomination or learning ideology, student composition, and (extra)curricular activities. About a quarter of a student’s primary school peers attend the same secondary school, resulting in a largely unfamiliar peer environment (Zwier et al., 2023).

Primary schools for the PRIMS project were selected using a stratified sample design. In total, 79 schools with 120 classes agreed to participate, resulting in a representative sample on region, level of urbanization, socio-economic composition, denomination, school size, and test scores at the school level. In total, 79 primary schools participated. All 2673 students in their final year(s) of primary education were invited to take part. In multi-grade classrooms where group 8 students shared a classroom with group 7 and/or 6 students, all students were invited to participate. Of the invited students, 1676 (62.7%) obtained active parental consent to participate. In total, 1634 students (61.1%) participated in the primary school measure, of which 1,408 were group 8 students. The remaining students did not participate, mainly because they were absent at the time. Students filled out the online survey during regular school hours under the supervision of their teacher, which took approximately 45 min. All 1288 group 8 students who participated in T1 and provided contact details were invited to participate in the secondary school wave (T2). In total, 758 students (58.9%) also participated in T2. The remaining students repeated a grade, did not provide valid contact details, or did not reply. Students filled out the online survey of approximately 15 min at home. PRIMS data were linked to administrative register data from NCO to calculate the share of co-transitioning primary school peers.

### Measures

#### Peer Attachment (T1, T2)

**S**tudents’ peer attachment was assessed using the trust subscale of the Inventory of Parent and Peer Attachment (IPPA; Armsden & Greenberg, 1987). The original scale was shortened from ten to three items, and items that focused on friends and parents were adapted to fit classmates (Zwier et al., 2023). The scale comprises three items: ‘My classmates accept me as I am’, ‘My classmates respect my feelings’, and ‘When I am angry about something, my classmates try to be understanding’. Students rated the items as 1 = *never* to 4 = *always*. The omega reliability for the measurement model of a univariate model using one time point was 0.785.

#### Loneliness (T1, T2)

Loneliness was measured using five items of the peer-related loneliness subscale of the Loneliness and Aloneness scale for Children and Adolescents (Lorijn et al., 2023; LACA; Marcoen et al., 1987). Sample items are ‘I feel left out by my friends’, ‘I feel sad because I have no friends’, and ‘I feel alone at school’. Students rated the items as 1 = *never* to 4 = *always*. The omega reliability for the measurement model of a univariate model using one time point was 0.841.

#### Victimization (T1, T2)

Victimization was measured by the question ‘Can you indicate how often you have been bullied at school in the past months?’, reflecting the global item of the traditional Olweus’ (1996) bully/victim questionnaire. Before asking this question, students watched an introductory clip that defines bullying as intentional, repeated harassment where the victim has problems defending themselves (Olweus, 1996; Zwier et al., 2023). The clip explains that bullying can take different forms, such as hitting or kicking, damaging belongings, gossiping, making fun of someone, or excluding someone, and that bullying can also take place online. Students could respond with 1 = *never* to 5 = *several times a week*.

#### Co-transitioning peers

Using data from administrative registers (NCO), the number of students who co-transitioned with the student from primary school to the same secondary school was calculated (see Zwier et al., 2023). The registers included information about the secondary school of *all* PRIMS students’ primary school grade mates, including those who did not participate in PRIMS themselves. Co-transitioning peers were defined as grade mates because classmates cannot be identified using NCO. Since Dutch primary schools are generally small, grade mates arguably know each other (e.g., the mean school cohort size in the sample was 30.4 students, with an *SD* of 16.2). The scores were transformed into proportions, accounting for differences in grade size. Scores thus range from 0–1, with 0 indicating no co-transitioning peers, and 1 indicating all primary school grade mates transitioned to the same secondary school. Additional analyses were conducted on same-gender co-transitioning peers as a proxy for friends given high gender homophily in pre-adolescents friendship networks (Zwier & Geven, 2023).

#### Academic performance (T2)

Academic performance was measured by students’ self-reported grades for Mathematics, Dutch, and English on their last report card. Grades ranged from 1 to 10, rounded to two decimals, with higher numbers indicating higher grades. As students are tracked in secondary school, this measure cannot capture student achievement across tracks (and curricula) but rather reflects how well students are doing in the track they were assigned to after primary school. The omega reliability for the measurement model was 0.433.

#### Gender

Students’ gender was controlled for, as previous research suggests that gender may be linked to both peer relationships and academic performance (Lorijn et al., 2022), and girls’ self-representations are more negatively affected by school transitions compared with boys (Schaffhuser et al., 2017). To assess gender, students indicated if they were a boy, girl, or other. Because only four students (0.5%) indicated to be ‘other’, ‘other’ was coded missing and gender as 0 = *boy*; 1 = *girl*.

### Analytic Strategy

#### Latent change score models

Latent change score (LSC) models were estimated to examine the impact of the baseline measure on the change in repeated measures. LCS models are a class of structural equation modeling, that combines strengths of path modeling and latent variable modeling (Kievit et al., 2018). Latent variables (multiple indicator models) were used instead of self-constructed scales to account for measurement errors in the observed items and to increase power and validity (Kievit et al., 2018). Change was examined over two time points with changes in y (y2 – y1) being modeled as latent change scores (Δy). In the multivariate model (which includes all three peer experience variables – attachment, loneliness, and victimization), effects of one variable at T1 (e.g., loneliness) on the change in another variable (e.g., attachment) were added (cross-domain coupling). The LCS models were estimated using the lavaan package in R version 4.3.2 (Rosseel, 2012). The hypotheses, methods, and analysis plan were preregistered at the Open Science Framework (OSF) after the data collection but prior to the analyses here: https://osf.io/y3q2w/.

LCS models assume multivariate normality of all observed variables, yet the measures for attachment, loneliness, and victimization are categorical (i.e., measured on Likert scales) and skewed. To handle this non-normality, the more robust diagonally weighted least squares (DWLS) estimator was used instead of the Maximum Likelihood (ML) default. A disadvantage of DWLS is that FIML cannot be applied to handle missing data. Therefore, complete cases on all items were needed to estimate a multiple-indicator model. Thus, listwise deletion was used to handle missing data. Of the 758 participants in both waves, 649 students had complete information on all items for attachment, loneliness, victimization, and gender. Data were mostly missing for (some items of) attachment at T2 (6.8%), loneliness at T2 (4.7%), and victimization at T2 (4.6%). The students included in model 1 (*n* = 649) did not differ from the nonresponding PRIMS students in their last year of primary school (*n* = 1408–649 = 759) on gender, attachment, loneliness, and victimization. In sum, 649 students (*M*_*ag*e_ T1 = 11.58, *SD* = 0.56; 53.6% girls) from 105 primary school classes and 77 primary schools were included in the main model. Strict measurement invariance over time was tested by constraining the factor loadings, measurement errors, and intercepts of the measurement models for loneliness and attachment to be equal over time. A comparison between the constrained and unconstrained model showed that the constraints did not significantly worsen the model fit for the univariate models of attachment (*X*^*2*^ (5, *N* = 649) = 5.43, *p* = 0.366) and loneliness (*X*^*2*^ (9, *N* = 649) = 11.84, *p* = 0.225), indicating measurement invariance. Therefore, the measurement models of both attachment and loneliness were constrained to be equal over time in all subsequent models that were estimated.

Model fit was assessed by testing the model fit with χ² tests and evaluating four fit measures: the Root Means Square Error of Approximation (RMSEA), the Comparative Fit Index (CFI), the Tucker-Lewis Index (TLI), and the standardized root mean squared residual (SRMR). The RMSEA should not exceed 0.08 and shows a good fit for values of ≤0.06. For CFI and TLI, ≥0.90 is considered acceptable, and ≥0.95 reflects a good model fit. An SRMR of ≤0.10 is considered acceptable, and ≤0.05 reflects a good fit (Little, 2013).

#### Hypotheses testing

**Fig. 1:**
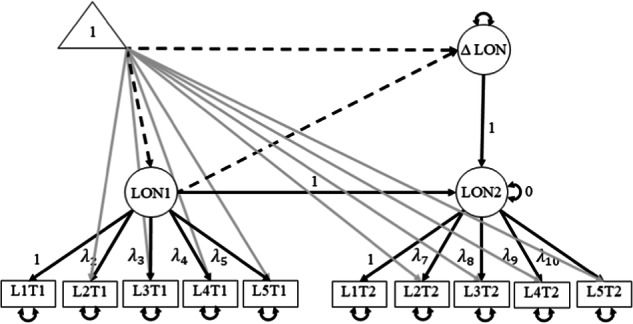
Path model of the univariate multiple indicator latent change score model for loneliness. The dashed paths are used to calculate the mean of the delta, the gray paths indicate the other paths from the constant for visual clarity. LON Loneliness, L1T1 Loneliness item 1 at T1, L1T1 Loneliness item 2 at T1, etc. Parameters for (residual) variances and covariances are omitted for visual clarity

Univariate LCS models were conducted to explore mean changes and tested the hypotheses in three multivariate multiple indicator LCS models. As a first step, three separate univariate LCS models for attachment, loneliness, and victimization to explore mean changes over the transition from primary to secondary school were estimated. Figure 1 shows the path model of the univariate multiple indicator latent change score model for loneliness, as an example. The latent construct for loneliness (LON) is measured at two time points (LON1 and LON2), each measured using five indicators (L1T1, etc.). The change in loneliness over the two time points is modeled as a latent change score (ΔLON). Means are included in the model by adding a constant term (value 1.0) as symbolized by the triangle in the path model. The mean changes were estimated by tracing all paths from the triangle to the latent change score (Δx), where the sum of all path values gives the estimated mean. For instance, the mean change for loneliness (*m*Δ LON) was estimated by summing the path from the triangle to ΔLON, and the path from the triangle to ΔLON via LON1, as indicated by the dashed paths in Fig. 1.

**Fig. 2:**
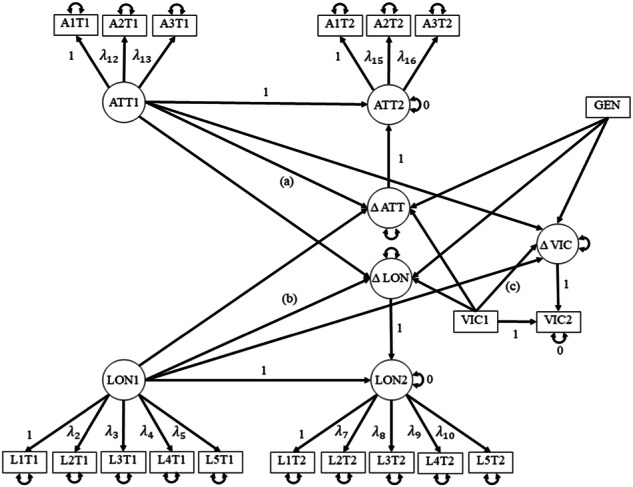
Path model of the multivariate multiple indicator latent change score model. LON Loneliness, L1T1 Loneliness item 1 at T1, L1T1 Loneliness item 2 at T1, etc., ATT Attachment, A1T1 Attachment item 1 at T1, etc., VIC Victimization, GEN Gender. Parameters for the constant, (residual) variances and covariances are omitted for visual clarity

As a second step, three multivariate LCS models, estimating changes in all three peer experiences simultaneously were conducted. In the first multivariate LCS model, hypotheses 1A, 1B, and 1C on how attachment, loneliness, and victimization in primary school relate to changes in these peer experiences over the transition were tested. Figure 2 shows the path model for the multivariate multiple indicator latent change score model with the constant being omitted for visual clarity. This model is an expansion from the univariate path model in Fig. 1. Hypothesis 1 was tested in paths (a), (b), and (c). Path (a) represents the effect of attachment in primary school (ATT1) on the change in attachment (ΔATT), path (b) represents the effect of loneliness in primary school (LON1) on the change in loneliness (ΔLON), and path (c) represents the effect of victimization in primary school (VIC1) on the change in victimization (ΔVIC). Hypotheses 1A, 1B and 1C were thus tested in the same multivariate model, meaning that estimates are conditional on the T1 measures of attachment, loneliness, victimization, and gender.

In the second multivariate LCS model, the proportion of co-transitioning peers was added as a predictor of the change. This way, Hypothesis 2 on whether the share of co-transitioning peers is negatively related to changes in attachment, loneliness, and victimization was tested. In total, 633 out of the 649 students (97.5%) were matched to co-transitioning peers data from the registers, leaving a sample size of 633 students for this model. The hypotheses were tested based on the mean deltas because this study was interested in the degree (rather than the direction) of changes in peer experiences. This means that if the mean delta of a peer experience was significantly positive (i.e., increased), it was expected on average a negative effect of co-transitioning peers on the peer experience, and vice versa.

In the third multivariate LCS model, academic performance was added as the outcome, while the change scores were defined as predictors. This way, it was examined to what extent changes in peer experiences predict academic performance in secondary school (Hypothesis 3). Academic performance was defined as a latent construct measured by three indicators. The sample size for model 3 was 559 because 559 out of the 649 students had complete cases on the indicators for academic performance. Means and covariances are provided for each of the three models separately because model estimation is needed to generate means for latent variables.

## Results

### (Dis)continuation of Peer Experiences

Average changes in attachment, loneliness, and victimization over the transition from primary to secondary school were explored by evaluating the mean delta’s (*m* Δ) of the univariate models of these variables. The model fit the data well for attachment (χ2(12, *N* = 649) = 6.74, *p* = 0.874, RMSEA = 0.000[0.000; 0.020], CFI = 1.000, TLI = 1.005, SRMR = 0.025) and loneliness (χ2(42, N = 649) = 32.09, *p* = 0.866, RMSEA = 0.000[0.000; 0.015], CFI = 1.000, TLI = 1.009, SRMR = 0.063). For victimization, only one item was used, leading to a fully identified model and, hence, a perfect fit. Results of the estimated means indicate that students slightly increased in attachment (*m*T1 = 3.37, *m*T2 = 3.45, *m*Δ = 0.082, se = 0.023, *p* < 0.01) and decreased in loneliness (*m*T1 = 1.29, *m*T2 = 1.21, *m*Δ = −0.074, se = 0.015, *p* < 0.01) between primary and secondary school. No significant average change in victimization between primary and secondary school was found (*m*T1 = 1.30, *m*T2 = 1.24, *m*Δ = −0.060, se = 0.042, *p* = 0.152). To gain more insight into the (dis)continuation of victimization, the percentage of students who were persistently victimized across the transition based on the single-item measure for victimization was calculated. Of the 649 students, 38 (5.9%) were victimized at least once or twice in the past months in primary- as well as secondary school.

Model 1 in Table 1 shows the results of the multivariate LSC model including attachment, loneliness, and victimization in primary school as predictors for change scores of these variables, controlled for gender. The model fit the data well (χ2(146, N = 649) = 108.18, *p* = 0.992, RMSEA = 0.000[0.000; 0.000], CFI = 1.000, TLI = 1.010, SRMR = 0.045). Hypotheses 1A, 1B and 1C were tested with the paths denoted as ‘(a)’, ‘(b)’, and ‘(c)’ in Fig. 1, of which the results are shown in Table 1 (model 1). Higher attachment in primary school related to a smaller change (i.e., a smaller increase or a decrease) in attachment over the transition from primary to secondary school (ATT 1 → Δ ATT = −0.638, se = 0.052, *p* < 0.001), in line with hypothesis 1 A. Specifically, the amount of change can be derived from the formula: Δ ATT = (ATT 1 → Δ ATT) * ATT 1 + I Δ ATT. With an intercept of the change in attachment (I Δ ATT) of 2.365, students who were not attached to peers in primary school experienced a larger increase in attachment than the average increase (Δ ATT(ATT 1 = 1) = 1.727), while students with high initial attachment experienced a small decrease in attachment (Δ ATT(ATT 1 = 4) = −0.187). These estimated effects are conditional on all other variables being constant.

**Table 1:** Results of three multivariate multiple-indicator latent change score models on change in attachment, loneliness, and victimization

	Model 1 (*n* = 649)		Model 2 (*n* = 633)		Model 3 (*n* = 559)	
	Est. (SE)	*p*	Est. (SE)	*p*	Est. (SE)	*p*
*m* Δ ATT	0.088 (0.025)	<0.001	0.088 (0.025)	0.001	0.093(0.026)	<0.001
I Δ ATT	2.365 (0.224)	<0.001	2.367 (0.225)	<0.001	2.527 (0.237)	<0.001
ATT 1 → Δ ATT (a)	−0.638 (0.052)	<0.001	−0.644 (0.053)	<0.001	−0.688 (0.056)	<0.001
LON 1 → Δ ATT	−0.139 (0.067)	0.039	−0.144 (0.068)	0.033	−0.213 (0.080)	0.008
VIC 1 → Δ ATT	−0.036 (0.040)	0.357	−0.035 (0.040)	0.383	0.016 (0.047)	0.728
GEN → Δ ATT	0.062 (0.043)	0.145	0.070 (0.043)	0.108	0.092 (0.045)	0.043
CTP → Δ ATT			0.035 (0.067)	0.603		
*m* Δ LON	−0.077 (0.016)	<0.001	−0.080 (0.016)	<0.001	−0.093 (0.017)	<0.001
I Δ LON	0.691 (0.145)	<0.001	0.727 (0.148)	<0.001	0.736 (0.154)	<0.001
LON 1 → Δ LON (b)	−0.647 (0.056)	<0.001	−0.643 (0.057)	<0.001	−0.674 (0.065)	<0.001
ATT 1 → Δ LON	−0.020 (0.031)	0.521	−0.014 (0.032)	0.659	−0.023 (0.034)	0.488
VIC 1 → Δ LON	0.058 (0.033)	0.078	0.053 (0.033)	0.110	0.042 (0.039)	0.289
GEN → Δ LON	0.037 (0.027)	0.170	0.035 (0.028)	0.200	0.045 (0.029)	0.124
CTP → Δ LON			−0.172 (0.041)	<0.001		
*m* Δ VIC	−0.060 (0.042)	0.152	−0.066 (0.043)	0.121	−0.064 (0.045)	0.153
I Δ VIC	0.657 (0.361)	0.069	0.647 (0.368)	0.079	0.795 (0.398)	0.046
VIC 1 → Δ VIC (c)	−0.755 (0.118)	<0.001	−0.758 (0.119)	<0.001	−0.766 (0.147)	<0.001
LON 1 → Δ VIC	0.171 (0.140)	0.221	0.184 (0.141)	0.193	0.145 (0.179)	0.419
ATT 1 → Δ VIC	0.035 (0.079)	0.654	0.049 (0.080)	0.537	0.014 (0.089)	0.871
GEN → Δ ATT	−0.049 (0.070)	0.482	−0.058 (0.072)	0.418	−0.067 (0.077)	0.382
CTP → Δ VIC			−0.118 (0.114)	0.299		
Δ ATT → AP					0.050 (0.081)	0.583
Δ LON → AP					0.150 (0.095)	0.113
Δ VIC → AP					−0.055 (0.058)	0.336
GEN → AP					0.283 (0.084)	0.001
Δ ATT~Δ LON	−0.102 (0.008)	<0.001	−0.100 (0.008)	<0.001	−0.101 (0.009)	<0.001
Δ ATT~Δ VIC	−0.055 (0.018)	0.002	−0.051 (0.018)	0.006	−0.056 (0.020)	0.004
Δ LON~Δ VIC	0.091 (0.017)	<0.001	0.087 (0.018)	<0.001	0.082 (0.019)	<0.001
R^2^ Δ ATT	30.0%		30.6%		32.7%	
R^2^ Δ LON	30.9%		31.5%		33.4%	
R^2^ Δ VIC	38.7%		39.1%		36.4%	
R^2^ AP					5.0%	

Higher initial loneliness was associated with decrease in the difference in loneliness over the transition (LON 1 → Δ LON = −0.647, se = 0.056, *p* < 0.001), in line with hypothesis 1B. Given this decrease in the difference, students who were more lonely in primary school experienced greater decreases in loneliness across the transition. With an I Δ LON of 0.691, the formula gives that students who were not lonely in primary school experienced small increases in loneliness (Δ LON(LON 1 = 1) = 0.044), whereas students who were more lonely experienced decreases in loneliness (Δ LON(LON 1 = 4) = −1.897). Higher victimization in primary school related to a decrease in the difference in victimization over the transition (VIC 1 → Δ VIC = −0.755, se = 0.118, *p* < 0.001), in line with hypothesis 1 C. Given this decrease in the difference, students who were more victimized in primary school experienced greater decreases in victimization across the transition. An I Δ VIC of 0.651 gives that all students decreased in victimization (Δ VIC(VIC 1 = 1) = −0.104), with greater decreases for students who were more victimized in primary school (Δ VIC(VIC 1 = 5) = −3.124).

The latent change scores of the multivariate model were similar to those from the univariate models, which implies that the intercepts and changes in one peer experience barely affect the mean change in the other peer experience. The degree of change in attachment was negatively related to the degree of change in loneliness and victimization, as shown by the covariances between the residuals and the estimated correlations (Δ ATT~Δ LON = −0.102, se = 0.008, *p* < 0.001, *r* = −0.477; Δ ATT ~ Δ VIC = −0.055, se = 0.018, *p* = 0.002, *r* = −0.179), and the degrees of change in loneliness and victimization were positively related (Δ LON ~ Δ VIC = 0.091, se = 0.017, *p* < 0.001, *r* = 0.278). Higher loneliness in primary school related to smaller increases in attachment over the transition (LON 1 → Δ ATT = −0.193, se = 0.067, *p* = 0.039). Gender did not relate to changes in attachment, loneliness, and victimization.

### Co-transitioning Peers

The number of co-transitioning peers was added to model 2 to test hypotheses 2A, 2B, and 2C on the effect of the percentage of students’ co-transitioning peers on changes in attachment, loneliness, and victimization. The model fit the data well (χ2(158, N = 633) = 114.74, *p* = 0.996, RMSEA = 0.000[0.000; 0.000], CFI = 1.000, TLI = 1.012, SRMR = 0.044). On average, 31.1% of students’ primary school peers co-transitioned into the same secondary school. Table 1 shows the results of Model 2. The estimated means of the delta’s and covariances between the delta’s are similar to those in Model 1. Having more co-transitioning peers (CTP) did not relate to changes in attachment (CTP → Δ ATT = −0.035, se = 0.067, *p* = 0.603) and victimization (CTP → Δ VIC = −0.118, se = 0.114, *p* = 0.299), contrary to hypotheses 2A and hypotheses 2C. Having more co-transitioning peers related to larger reductions in loneliness (CTP → Δ LON = −0.127, se = 0.041, *p* < 0.001). It was expected that having co-transitioning peers would relate to fewer changes in loneliness over the transition, but the findings indicated that there are *more* changes in loneliness when there are more co-transitioning peers. Therefore, the findings are not in line with hypothesis 2B.

The number of co-transitioning peers was replaced with the number of co-transitioning same-gender peers as a proxy for friends in an additional model. On average, 32.0% of students’ primary school peers with the same gender co-transitioned into the same secondary school. The model had acceptable fit (χ2(178, N = 633) = 487.95, *p* < 0.001, RMSEA = 0.052 [0.047; 0.058], CFI = 0.936, TLI = 0.924, SRMR = 0.077). The results were similar to model 3. Having more co-transitioning peers with the same gender did not relate to changes in attachment (CTP → Δ ATT = 0.019, se = 0.064, *p* = 0.761) and victimization (CTP → Δ VIC = −0.085, se = 0.108, *p* = 0.433), and related to larger reductions in loneliness (CTP → Δ LON = −0.111, se = 0.038, *p* = 0.004).

### Academic Performance

In Model 3, the changes in attachment, loneliness, and victimization were regressed on academic performance to test hypothesis 4 on the extent to which changes in peer relationships relate to higher academic performance. The model had acceptable fit (χ2(199, N = 559) = 160.28, *p* = 0.980, RMSEA = 0.000[0.000; 0.000], CFI = 1.000, TLI = 1.012, SRMR = 0.047). Table 1 shows the results of Model 3. The estimated latent mean of academic performance shows that on average, students scored 7.26 out of 10. Results indicate that changes in attachment (Δ ATT → AP = 0.050, se = 0.081, *p* = 0.538), loneliness (Δ LON → AP = 0.150, se = 0.095, *p* = 0.113), or victimization (Δ VIC → AP = −0.055, se = 0.058, *p* = 0.336) over the transition from primary to secondary school had no relevant and significant effect on academic performance (AP) in secondary school. This does not support hypotheses 4A, 4B, and 4C. Girls had a higher academic performance compared to boys (GEN → AP = 0.283, se = 0.084, *p* = 0.001).

## Discussion

Prior studies on peer relationships in school transitions neglected heterogeneity, or did not examine the relation with academic performance in secondary school. This study investigated to what extent attachment, loneliness, and victimization (dis)continued over the transition from primary to secondary school. Beyond studying average changes, this study examined to what extent students’ peer experiences in primary school and the amount of change in the peer group related to (dis)continuations, and to what extent (dis)continuations in experiences related to academic performance in secondary school. Students, on average, became more attached to peers and less lonely following the transition to secondary school. No differences in victimization between primary and secondary school were found. The degree of change in loneliness was positively related to the degree of change in victimization. Students who experienced a greater decrease in loneliness also experienced a greater decrease in victimization, and vice versa. Students who were more attached to peers in primary school had smaller increases in attachment over the transition or even experienced slight decreases in attachment. Students who were lonelier or victimized in primary school experienced greater reductions in loneliness and victimization across the transition, respectively. Students who had more co-transitioning peers experienced greater reductions in loneliness. Changes in peer experiences over the transition did not relate to academic performance in secondary school.

### (Dis)continuation of Peer Experiences

Students’ lower levels of loneliness following a school transition were in line with some previous studies (Barber & Olsen, 2004; Lorijn et al., 2023). Yet, the results show a more positive view of school transitions than most previous studies (Jindal‐Snape et al., 2020). Entering a new peer group may provide a “fresh start” in terms of peer relationships. Based on the stage-environment fit theory (Eccles et al., 1997), the secondary school environment in the Netherlands may match adolescents’ need for peer relationships. This transition to a new peer group provides an opportunity to improve students’ peer experiences and thus promote adolescents’ social adjustment. Secondary schools provide a wider range of peers compared to primary school, including not only classmates but also older peers and peers in tutor groups (Symonds & Hargreaves, 2016). Particularly in countries characterized by a dense clustering of schools and considerable freedom of choice, such as the Netherlands, students enjoy greater flexibility in choosing a secondary school that matches their preferences. Specifically, while students must adhere to their track recommendations when choosing a school, they have considerable freedom within these parameters. Financial and geographic barriers to school choice are minimal and school autonomy is high (Zwier et al., 2023). This allows secondary schools to differentiate themselves by specializing in certain areas, such as religion, languages, arts, or sports to attract a subgroup of students. In result, this gives students the autonomy to choose a secondary school that best fits their desires, leading to better adjustment following the transition (De Moor & Branje, 2023). This freedom of education already exists in primary education, but students may exercise greater autonomy in selecting a secondary school that suits their preferences rather than solely relying on their parents’ choice. In conjunction with ability tracking, secondary school peers may therefore be more homogenous in terms of level of education, interests, norms, values, and family background. Together, this may create a school environment in which it is easier to connect with like-minded peers and fit into the peer group.

These social benefits of school transitions may be temporary. For instance, a study on the transition to vocational education found that 10% of students experienced a temporary increase in depressive symptoms, and another 10% of students experience a temporary decrease in depressive symptoms after the transition, returning to their original level two years later (Visscher et al., 2024). This suggests that students’ socio-emotional wellbeing may not be linear around school transitions and that changes may be temporary. However, another study found that students’ social position in the new peer environment in secondary school was determined at the start of secondary education, and did not change over the next two years (De Vries et al., 2021). Future studies would extend the findings of this study by assessing students’ peer experiences throughout secondary school.

### Primary School Peer Experiences

The extent to which students’ peer experiences changed over the transition from primary to secondary school, depended on their peer experiences in primary school, in line with the hypotheses. Students with positive peer experiences in primary school were less likely to socially gain from transitioning to a new peer group. Conversely, students with negative peer experiences in primary school were more likely to experience a “fresh start” in terms of peer relationships in secondary school. Thus, students who had more extreme scores in primary school became more average in secondary school, raising the question to what extent regression to the mean effects are at play. Whereas more traditional models (e.g., regression models or cross-lagged panel models) assume stationarity, LCS models accommodate natural change by including a slope latent variable (similar to a paired t-test; Coman et al., 2013; King et al., 2006). However, although regression to the mean effects may be a natural occurrence, in the case of Lords Paradox it may lead to biased results even in LCS models (Sorjonen et al., 2022). Lords Paradox occurs particularly for variables that predict the change in y other than the baseline measure of y, in which case corrections are recommended (Sorjonen et al., 2022). This is not the case when the change in y is predicted by the baseline measure of y, because there is no spurious relation between x and the change in y via the baseline measure of y. Moreover, a natural regression to the mean effect can be assumed for the results because there was a mean increase in attachment and decrease in loneliness. Both attachment (*m*T1 = 3.37, scaled from 1–4) and loneliness (*m*T1 = 1.29, scaled from 1–4) were skewed at baseline, suggesting that an artificial regression to the mean effect would result in a mean decrease in attachment and increase in loneliness.

The findings are in line with previous studies finding that for most students who were victimized in primary school, victimization did not persist in secondary school (Låftman et al., 2024; Pellegrini et al., 2010). This may be explained by the reorganization of the social hierarchy in a new peer context (Farmer et al., 2011). Students who were at the bottom of the social hierarchy in primary school may improve their status in a new peer context. Whereas the social lives of most students improved across the transition, particularly for those with more negative peer experiences in primary school, this is not the case for all students. Specifically, students who were more lonely in primary school generally increased less in attachment over the transition. Possibly, whereas students who were lonely in primary school may decrease in feelings of loneliness, they may lack the social skills and self-esteem to develop close social relationships in the new peer group. Furthermore, roughly one student per classroom (5.9% of all students) experienced persistent victimization across the transition from primary to secondary school. Possibly, these students lack the social skills to improve their social position in the new peer group. Persistent victims may be at particular risk of lowered psychological and school adjustment (Huitsing et al., 2019), and may therefore be the focus of future research.

The degree of change in one peer experience was related to the degree of change in another peer experience. Students who became more attached to peers in secondary school tended to become less lonely and less victimized, and vice versa. Students who experienced greater decreases in loneliness were more likely to experience decreases in victimization, and vice versa. This underscores the interconnectedness of different peer experiences and suggests that improvements in one peer experience may coincide with improvements in other peer experiences. Future research could apply LCS models to further examine the effects of coupling across domains (Kievit et al., 2018).

### Co-transitioning Peers

On average, roughly a third of students’ primary school peers transitioned to the same secondary school. Students with more co-transitioning peers experienced greater reductions in loneliness in secondary school, but no differences in changes in attachment and victimization, contrary to the expectations. Thus, rather than students having similar levels of loneliness in secondary school when more peers were familiar, these students benefitted from having more familiar peers in terms of loneliness. Likely, a more familiar peer group protects against feeling lonely (Benner, 2011). Moreover, familiar peers may provide transitional support during challenging school transitions (Zwier et al., 2023), lowering feelings of loneliness.

No adverse effects of having more co-transitioning peers were found, with no evidence of harm for students who were less attached or more victimized in primary school to transition to secondary school with more primary school peers. These findings were contrary to the expectations that having more co-transitioning peers would result in more stable peer experiences. It may be that the extent to which peer experiences remain stable depends on which primary school peers co-transition rather than how many peers co-transition. For instance, peer attachment may be particularly stable when students’ friends co-transition, whereas victimization may be particularly persistent when a victims’ bully co-transitions. Moreover, the effects of co-transitioning peers may be smaller in the Dutch context because students from multi-track primary schools are generally tracked within classes in secondary school (i.e., within-school ability grouping), meaning that co-transitioning peers do not always end up in the same classroom. In addition, it may be advantageous to transition with a subset of primary school peers, rather than with either none or many peers. This would facilitate the maintenance of existing peer relationships while providing opportunities to form new ones (Langenkamp, 2009). Future studies would add to the literature by examining the optimal composition of secondary school classes including a proportion of unfamiliar peers to shuffle the existing hierarchy and a proportion of familiar peers to further reduce feelings of loneliness.

### Academic Consequences of Changing Peer Experiences

Changes in peer experiences over the transition did not relate to academic performance in secondary school, contrary to the expectations. Students’ peer relationships and academic performance in secondary school were measured roughly five months after the students transitioned to secondary education. This may be too soon to see a positive effect of improved peer relationships on students’ academic performance. Positive peer relationships may enhance students’ self-esteem, self-efficacy, engagement, and motivation, improving their academic performance (Kiuru et al., 2020; Wentzel et al., 2021). However, these characteristics may take time to improve, particularly when negative peer experiences in primary school have long-lasting effects. Moreover, the relation between the change in peer experience and academic performance may depend on students’ initial peer experience. For instance, an increase in attachment may only matter in terms of academic performance for students with low attachment in primary school, and less for those who were already more attached in primary school. However, following this reasoning, a smaller decrease or larger increase in victimization or loneliness would be reflected in lower performance, yet this was not found.

### Strengths and Limitations

This study has several strengths. Beyond examining average changes in attachment, loneliness, and victimization over the transition from primary to secondary school, it was studied how students’ social position and the number of co-transitioning peers related to this change, and to what extent changes in peer experiences related to academic performance in secondary school. In doing so, this study overcame methodological shortcomings in previous studies. Whereas school transitions are longitudinal by definition, only 41% of the studies on peer relationships in school transitions were quantitative longitudinal studies (Jindal‐Snape et al., 2020). Recent data collected in primary and secondary schools with a sufficient sample size were used to better capture this longitudinal nature. Moreover, the data were matched to register data from the Netherlands Cohort Study on Education (NCO) to incorporate the number of co-transitioning peers. Latent Change Score models were used to best analyze change scores. Lastly, whereas most previous studies on school transitions were conducted in the U.S.A. or U.K. (Jindal‐Snape et al., 2020), this study adds to the literature by using data from the Netherlands.

Despite these strengths, this study has some limitations, particularly for the results on academic performance. Only one measure of self-reported academic performance in secondary school roughly five months after students transitioned was available, limiting the possibility to examine the long-term effects of changes in peer experiences on students’ academic performance. In addition, controlling for students’ academic performance in primary school was not possible. As students are tracked in secondary school, students’ achievement in primary school (across tracks) is not expected to have a linear relation with students’ achievement in secondary school (within tracks and curricula). For instance, scoring an eight out of ten shows a different level of achievement on the lowest than on the highest track of secondary school. The measure thus reflects how well students do in the track they were assigned to in secondary school. Future studies may aim to follow students throughout secondary school to examine long-term relations between (changed) peer experiences and performance. Moreover, further research could increase their sample size to examine different groups based on students’ social position in primary school and ability track in secondary school. This would allow, for example, to examine whether changes in peer relationships vary as a function of track. A larger sample may also allow to examine moderation effects or differences between groups by students’ initial peer experiences.

### Practical Implications

The findings of this study have implications for school practice and interventions. The findings show that school transitions may improve students’ social lives. Whereas students with positive peer experiences in primary school socially benefitted less from transitioning to a new peer group, students with more negative peer experiences in primary school benefitted most. Thus, changing the social structure of the class may shuffle the social hierarchy which can provide a “fresh start” to students who need it most. With students worrying most about their peer relationships as they transition to secondary school (Jindal‐Snape et al., 2020), teachers may highlight the positive aspects of school transitions for peer relationships to relieve these worries. Although it was not found that school transitions harm students’ social lives, some students have persistent negative peer experiences across school transitions. Roughly one student per classroom (5.9% of all students) was persistently victimized over the transition from primary to secondary school in this study. These students should be the focus of school practice and interventions because they may be at particular risk of lowered psychological and school adjustment (Huitsing et al., 2019). There is a debate on to what extent secondary schools should be informed if students were victimized in primary school as this may result in stigmatizing, lower teacher expectations, and self-fulfilling prophecies (Rosenthal & Jacobson, 1968). However, secondary schools would need to be informed about students’ victimization history in primary school to identify students who are at risk for persistent victimization to target these students for intervention. Future studies and school practice could focus on how best to exchange this knowledge between primary and secondary schools to target the right students in a professional way that avoids stigmatization of these students.

## Conclusion

School transitions pose significant challenges for students’ peer relationships. Previous studies neglected individual differences in changing peer experiences, or did not examine the relation between changes in peer experiences and academic performance in secondary school. This study examined how students’ social position and the share of co-transitioning peers related to changes in attachment, loneliness, and victimization over the transition from primary to secondary school, and to what extent changes in peer experiences related to academic performance in secondary school. The findings show that school transitions may not only harm students’ peer relationships. Students, on average, became more attached to peers and less lonely, and did not change in victimization following the transition to secondary school. Particularly students with more negative peer experiences in primary school were shown to have a “fresh start” in secondary school. Students who improved more in one peer experience, also showed greater improvement in other peer experiences. Having more primary school friends who transition to the same secondary school was beneficial in terms of loneliness. Changes in peer experiences were not related to students’ academic performance in the first year of secondary school. Teachers can reduce students’ worries about school transitions by emphasizing these social benefits.

## Data Availability

PRIMS data will be made publicly available for researchers under certain conditions in 2024, see: Zwier, Dieuwke; Lorijn, Sofie J.; van den Brink, Eline; Bol, Thijs; Geven, Sara; van de Werfhorst, Herman G.; Engels, Maaike C.; Veenstra, René, 2023, "Peer Relations in the Transition from Primary to Secondary education (PRIMS)", 10.34894/U6XDT0, DataverseNL, V1. NCO data are accessible for statistical and scientific research under conditions. For more information, and to request access to the data via Statistics Netherlands (CBS), see: CBS: https://www.cbs.nl/en-gb/our-services/customised-services-microdata/microdata-conducting-yourown-research) and NCO: https://www.nationaalcohortonderzoek.nl/onderzoek.

## References

[CR1] Almeida, D. M., & Wong, J. D. (2009). Life transitions and daily stress processes. In G. H. Elder & J. Z. Giele (Eds.), *The craft of life course research* (pp. 141–162). The Guilford Press.

[CR2] Armsden, G. C., & Greenberg, M. T. (1987). The inventory of parent and peer attachment: Individual differences and their relationship to psychological well-being in adolescence. *Journal of Youth and Adolescence*, *16*(5), 427–454. 24277469 10.1007/BF02202939

[CR3] Bagwell, C. L., & Bukowski, W. M. (2018). Friendship in childhood and adolescence: Features, effects, and processes. In W. M. Bukowski, B. Laursen, & K. H. Rubin (Eds.), *Handbook of peer interactions, relationships, and groups* (2nd ed., pp. 371–390). Guilford Press.

[CR4] Barber, B. K., & Olsen, J. A. (2004). Assessing the transitions to middle and high school. *Journal of Adolescent Research*, *19*(1), 3–30. 10.1177/0743558403258113

[CR5] Benner, A. D. (2011). The transition to high school: Current knowledge, future directions. *Educational Psychology Review*, *23*(3), 299. 21966178 10.1007/s10648-011-9152-0 PMC3182155

[CR6] Benner, A. D., Boyle, A. E., & Bakhtiari, F. (2017). Understanding students’ transition to high school: Demographic variation and the role of supportive relationships. *Journal of Youth and Adolescence*, *46*(10), 2129–2142. 28776119 10.1007/s10964-017-0716-2 PMC5693765

[CR7] Benner, A. D., & Graham, S. (2009). The transition to high school as a developmental process among multiethnic urban youth. *Child Development*, *80*(2), 356–376. 19466997 10.1111/j.1467-8624.2009.01265.x

[CR8] Bowlby, J. (1973). Separation, anxiety and anger. In *Attachment and Loss: Volume II* (pp. 1–429). Hogarth Press and the Institute of Psycho-Analysis.

[CR56] Coman, E. N., Picho, K., McArdle, J. J., Villagra, V., Dierker, L., & Iordache, E. (2013). The paired t-test as a simple latent change score model. *Frontiers in psychology*, *4*, 738. 24124419 10.3389/fpsyg.2013.00738 PMC3794455

[CR9] De Moor, E. L., & Branje, S. (2023). Examining secondary school choice processes as a predictor of adjustment after the school transition. *Journal of Research on Adolescence*, *33*(1), 251–268. 36200304 10.1111/jora.12801

[CR10] De Vries, E., Kaufman, T. M. L., Veenstra, R., Laninga-Wijnen, L., & Huitsing, G. (2021). Bullying and victimization trajectories in the first years of secondary education: Implications for status and affection. *Journal of Youth and Adolescence*, 1–12. 10.1007/s10964-020-01385-w PMC8416874 33464443

[CR11] Deci, E. L., & Ryan, A. M. (2008). Self-determination theory: A macrotheory of human motivation, development, and health. *Canadian Psychology/Psychologie Canadienne*, *49*(3), 182. 10.1037/a0012801

[CR12] Eccles, J. S., Midgley, C., Wigfield, A., Buchanan, C. M., Reuman, D., Flanagan, C., & Mac Iver, D. (1997). Development during adolescence: The impact of stage–environment fit on young adolescents’ experiences in schools and in families (1993). In J. M. Notterman (Ed.), *The evolution of psychology: Fifty years of the American Psychologist*. American Psychological Association. 10.1037/10254-034. 10.1037//0003-066x.48.2.90 8442578

[CR14] Evangelou, M., Taggart, B., Sylva, K., Melhuish, E., Sammons, P., & Siraj-Blatchford, I. (2008). *Effective pre-school, primary and secondary education 3-14 project (EPPSE 3-14): What makes a successful transition from primary to secondary school?*

[CR15] Farmer, T. W., Hamm, J. V., Leung, M.-C., Lambert, K., & Gravelle, M. (2011). Early adolescent peer ecologies in rural communities: Bullying in schools that do and do not have a transition during the middle grades. *Journal of Youth and Adolescence*, *40*(9), 1106–1117. 21667294 10.1007/s10964-011-9684-0

[CR16] Felmlee, D., McMillan, C., Inara Rodis, P., & Osgood, D. W. (2018). Falling behind: Lingering costs of the high school transition for youth friendships and grades. *Sociology of Education*, *91*(2), 159–182. 10.1177/0038040718762136

[CR17] Haelermans, C., Huijgen, T., Jacobs, M., Levels, M., van der Velden, R., van Vugt, L., & van Wetten, S. (2020). Using data to advance educational research, policy, and practice: Design, content, and research potential of the Netherlands Cohort Study on Education. *European Sociological Review*, *36*(4), 643–662. 10.1093/esr/jcaa027

[CR18] Heinrich, L. M., & Gullone, E. (2006). The clinical significance of loneliness: A literature review. *Clinical Psychology Review*, *26*(6), 695–718. 16952717 10.1016/j.cpr.2006.04.002

[CR19] Huitsing, G., Lodder, G. M. A., Oldenburg, B., Schacter, H. L., Salmivalli, C., Juvonen, J., & Veenstra, R. (2019). The healthy context paradox: Victims’ adjustment during an anti-bullying intervention. *Journal of Child and Family Studies*, *28*(9), 2499–2509. 10.1007/s10826-018-1194-1

[CR20] Huitsing, G., Veenstra, R., Sainio, M., & Salmivalli, C. (2012). “It must be me” or “It could be them?”: The impact of the social network position of bullies and victims on victims’ adjustment. *Social Networks*, *34*(4), 379–386. 10.1016/j.socnet.2010.07.002

[CR21] Jindal‐Snape, D., Hannah, E. F. S., Cantali, D., Barlow, W., & MacGillivray, S. (2020). Systematic literature review of primary‒secondary transitions: International research. *Review of Education*, *8*(2), 526–566. 10.1002/rev3.3197

[CR23] Kievit, R. A., Brandmaier, A. M., Ziegler, G., van Harmelen, A.-L., de Mooij, S. M., Moutoussis, M., Goodyer, I. M., Bullmore, E., Jones, P. B., & Fonagy, P. (2018). Developmental cognitive neuroscience using latent change score models: A tutorial and applications. *Developmental Cognitive Neuroscience*, *33*, 99–117. 29325701 10.1016/j.dcn.2017.11.007 PMC6614039

[CR57] King, L. A., King, D. W., McArdle, J. J., Saxe, G. N., Doron-LaMarca, S., & Orazem, R. J. (2006). Latent difference score approach to longitudinal trauma research. *Journal of Traumatic Stress: Official Publication of the International Society for Traumatic Stress Studies*, *19*(6), 771–785. 10.1002/jts.20188 17195976

[CR24] Kingery, J. N., Erdley, C. A., & Marshall, K. C. (2011). Peer acceptance and friendship as predictors of early adolescents’ adjustment across the middle school transition. *Merrill-Palmer Quarterly*, *1982-*, 215–243. 10.1353/mpq.2011.0012

[CR25] Kinney, D. A. (1993). From nerds to normals: The recovery of identity among adolescents from middle school to high school. *Sociology of Education*, 21–40.

[CR26] Kiuru, N., Wang, M.-T., Salmela-Aro, K., Kannas, L., Ahonen, T., Hirvonen, R., & Buehner, R. E. (2020). Associations between Adolescents’ Interpersonal Relationships, School Well-being, and Academic Achievement during Educational Transitions. *Journal of Youth and Adolescence*, *49*(5), 1057–1072. 31893326 10.1007/s10964-019-01184-y PMC7182546

[CR27] Kretschmer, T., Veenstra, R., Branje, S., Reijneveld, S. A., Meeus, W. H. J., Deković, M., Koot, H. M., Vollebergh, W. A. M., & Oldehinkel, A. J. (2018). How competent are adolescent bullying perpetrators and victims in mastering normative developmental tasks in early adulthood? *Journal of Abnormal Child Psychology*, *46*(1), 41–56. 28593601 10.1007/s10802-017-0316-3 PMC5770496

[CR28] Låftman, S. B., Grigorian, K., Lundin, A., Östberg, V., & Raninen, J. (2024). Bullying experiences before and after the transition from lower to upper secondary school: Associations with subsequent mental health in a Swedish cohort. *BMC Public Health*, *24*(1), 27. 38166802 10.1186/s12889-023-17443-4 PMC10762947

[CR29] Langenkamp, A. G. (2009). Following different pathways: Social integration, achievement, and the transition to high school. *American Journal of Education*, *116*(1), 69–97. 20664813 10.1086/605101 PMC2906826

[CR30] Lessard, L. M., & Juvonen, J. (2022). The academic benefits of maintaining friendships across the transition to high school. *Journal of School Psychology*, *92*, 136–147. 35618366 10.1016/j.jsp.2022.03.005

[CR31] Little, T. D. (2013). *Longitudinal structural equation modeling*. Guilford press.

[CR32] Lorijn, S. J., Engels, M. C., Huisman, M., & Veenstra, R. (2022). Long-Term Effects of Acceptance and Rejection by Parents and Peers on Educational Attainment: A Study from Pre-Adolescence to Early Adulthood. *Journal of Youth and Adolescence*, *51*(3), 540–555. 34609673 10.1007/s10964-021-01506-z PMC8881433

[CR33] Lorijn, S. J., Laninga‐Wijnen, L., Engels, M. C., Lodder, G. M. A., & Veenstra, R. (2023). The Development of Adolescents’ Loneliness during the COVID-19 Pandemic: The Role of Pre-Pandemic Peer Status and During-Pandemic Contacts with Friends. *PloS One*, *18*(5), e0286085. 10.1371/journal.pone.0286085. 37235574 10.1371/journal.pone.0286085 PMC10218743

[CR34] Marcoen, A., Goossens, L., & Caes, P. (1987). Lonelines in pre-through late adolescence: Exploring the contributions of a multidimensional approach. *Journal of Youth and Adolescence*, *16*(6), 561–577. 24277491 10.1007/BF02138821

[CR35] Olweus, D. (1993). *Bullying at school: What we know and what we can do*. Blackwell.

[CR36] Olweus, D. (1996). Revised Olweus bully/victim questionnaire. *British Journal of Educational Psychology*. APA PsycTests. 10.1037/t09634-000.

[CR37] Pellegrini, A. D., Long, J. D., Solberg, D., Roseth, C., Dupuis, D., Bohn, C., & Hickey, M. (2010). Bullying and social status during school transitions. *Handbook of Bullying in Schools: An International Perspective*, 199–210.

[CR38] Perlman, D., & Peplau, L. A. (1981). Toward a social psychology of loneliness. *Personal Relationships*, *3*, 31–56.

[CR39] Poorthuis, A. M. G., Thomaes, S., van Aken, M. A. G., Denissen, J. J. A., & Orobio de Castro, B. (2014). Dashed hopes, dashed selves? A sociometer perspective on self‐esteem change across the transition to secondary school. *Social Development*, *23*(4), 770–783. 10.1111/sode.12075

[CR40] Rosenthal, R., & Jacobson, L. (1968). Pygmalion in the classroom. *The Urban Review*, *3*(1), 16–20. 10.1007/BF02322211

[CR41] Ryan, A. M., & Shin, H. (2018). Peers, academics and teachers. In W. M. Bukowski, B. Laursen, & K. H. Rubin (Eds.), *Handbook of peer interactions, relationships and groups* (pp. 637–646). Guilford Press New York, NY.

[CR55] Rosseel, Y. (2012). lavaan : An R Package for Structural Equation Modeling. *Journal of Statistical Software*, *48*, 1–36. 10.18637/jss.v048.i02

[CR42] Schacter, H. L., Hoffman, A. J., Ehrhardt, A., & Bakth, F. (2022). Peer victimization, schooling format, and adolescent internalizing symptoms during the COVID-19 pandemic: Between-and within-person associations across ninth grade. *Development and Psychopathology*, *35*(2), 823–837. 10.1017/S0954579422000074 35152917

[CR43] Schaffhuser, K., Allemand, M., & Schwarz, B. (2017). The development of self-representations during the transition to early adolescence: The role of gender, puberty, and school transition. *The Journal of Early Adolescence*, *37*(6), 774–804. 10.1177/0272431615624841

[CR58] Sorjonen, K., Melin, B., & Nilsonne, G. (2022). Lord’s paradox in latent change score modeling: An example involving facilitating longitudinal effects between intelligence and academic achievement. *Personality and Individual Differences*, *189*, 111520. 10.1016/j.paid.2022.111520

[CR44] Symonds, J., & Hargreaves, L. (2016). Emotional and motivational engagement at school transition: A qualitative stage-environment fit study. *The Journal of Early Adolescence*, *36*(1), 54–85. 10.1177/0272431614556348

[CR45] Temkin, D. A., Gest, S. D., Osgood, D. W., Feinberg, M., & Moody, J. (2018). Social network implications of normative school transitions in non-urban school districts. *Youth & Society*, *50*(4), 462–484. 29628532 10.1177/0044118X15607164 PMC5886351

[CR46] Veenstra, R., & Laninga-Wijnen, L. (2023). The prominence of peer interactions, relationships, and networks in adolescence and early adulthood. In C. G. Crockett & J. E. Schulenberg (Eds.), *APA handbook of adolescent and young adult development*. (pp. 225–241). American Psychological Association. 10.1037/0000298-014.

[CR47] Virtanen, T. E., Vasalampi, K., Torppa, M., Lerkkanen, M.-K., & Nurmi, J.-E. (2019). Changes in students’ psychological well-being during transition from primary school to lower secondary school: A person-centered approach. *Learning and Individual Differences*, *69*, 138–149. 10.1016/j.lindif.2018.12.001

[CR48] Visscher, A. H., Sijtsema, J., Van Roekel, E., & Bogaerts, S. (2024). Developmental trajectories of adolescents’ depressive symptoms surrounding the transition from pre-vocational to vocational education. *(Under Review)*. 10.17605/OSF.IO/M463N.

[CR49] Wentzel, K. R., Jablansky, S., & Scalise, N. R. (2021). Peer social acceptance and academic achievement: A meta-analytic study. *Journal of Educational Psychology*, *113*, 157–180. 10.1037/edu0000468. 10.1037/edu0000468

[CR50] Zwier, D., & Geven, S. (2023). Knowing me, knowing you: Socio-economic status and (segregation in) peer and parental networks in primary school. *Social Networks*, *74*, 127–138. 10.1016/j.socnet.2023.03.003

[CR51] Zwier, D., Geven, S., Bol, T., & Van de Werfhorst, H. G. (2023). Let’s Stick Together: Peer Effects in Secondary School Choice and Variations by Student Socio-Economic Background. *European Sociological Review*, *39*(1), 67–84. 10.1093/esr/jcac033

[CR52] Zwier, D., Lorijn, S. J., Van den Brink, E., Bol, T., Geven, S., Van de Werfhorst, H. G., Engels, M. C., & Veenstra, R. (2023). *PRIMS, Data collection report and codebook, version 1.0*. DataverseNL. 10.34894/U6XDT0.

